# Supplementary material for: Family-based selection: an efficient method for increasing phenotypic variability
Source: G3 (Bethesda). 2025 Jul 18;15(10):jkaf165. doi: 10.1093/g3journal/jkaf165 (PMC12506656; doi:10.1093/g3journal/jkaf165)
Supplement: jkaf165_Supplementary_Data [file jkaf165_Supplementary_Data.zip › Figure_S10_G3-2025-405909.pdf]

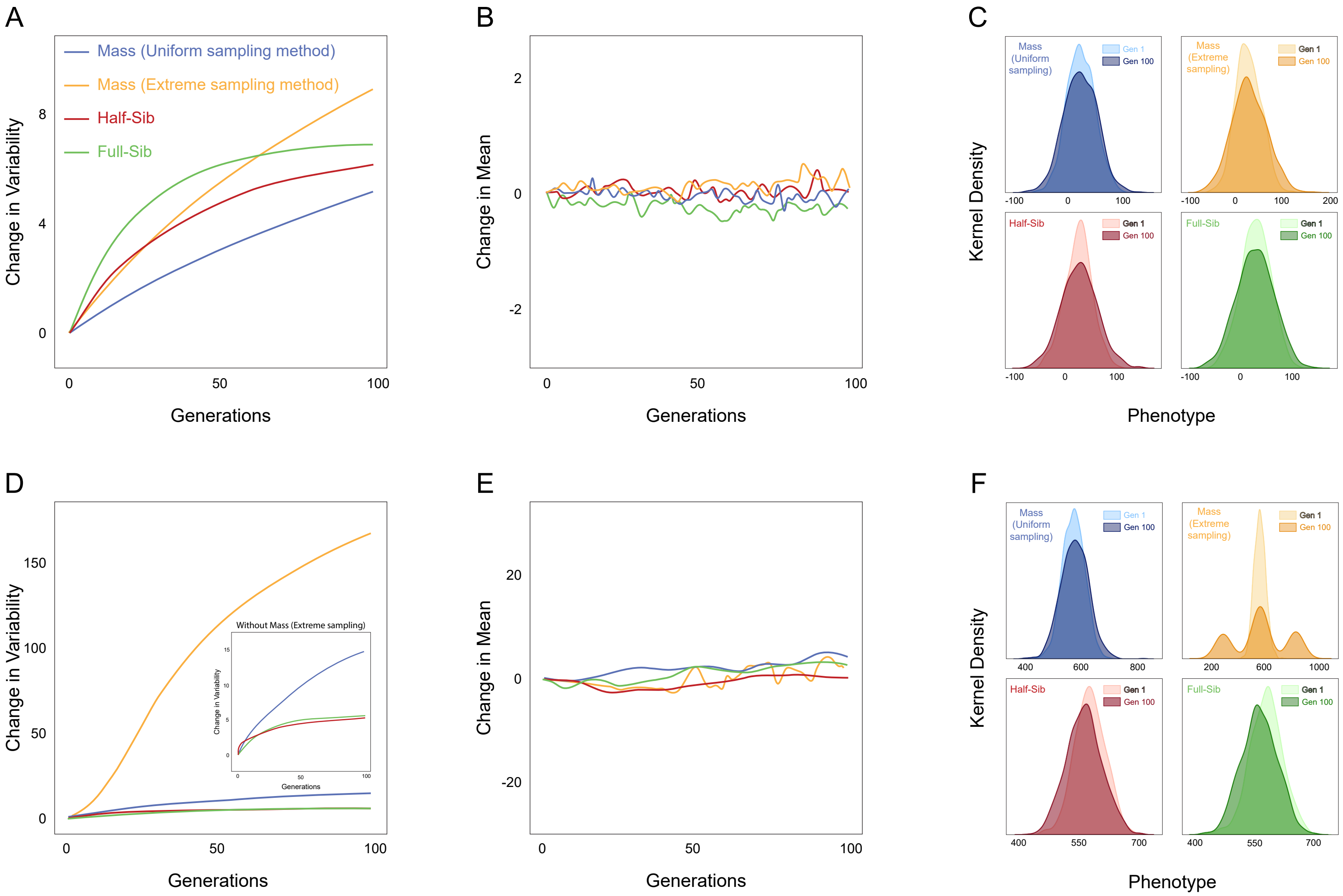

Figure S10: Selection response when both mean and variability are heritable

Panels (A-C) assume trait mean and variability have independent sites with effects drawn from identical distributions. Panels (D-F) assume 15x larger site effects for the mean of the trait versus its variability. A,D) Average change in variability as a function of the number of generations of selection. Inset in D magnifies the responses to selection regimes other than extreme mass selection. (B,E) Average change in mean as a function of number of generations of variability selection. (C,F) Example kernel density estimates of the distributions of trait values at generations 1 and 100 across selection regimes
