# Supplementary material for: Family-based selection: an efficient method for increasing phenotypic variability
Source: G3 (Bethesda). 2025 Jul 18;15(10):jkaf165. doi: 10.1093/g3journal/jkaf165 (PMC12506656; doi:10.1093/g3journal/jkaf165)
Supplement: jkaf165_Supplementary_Data [file jkaf165_Supplementary_Data.zip › Figure_S1_G3-2025-405909.pdf]

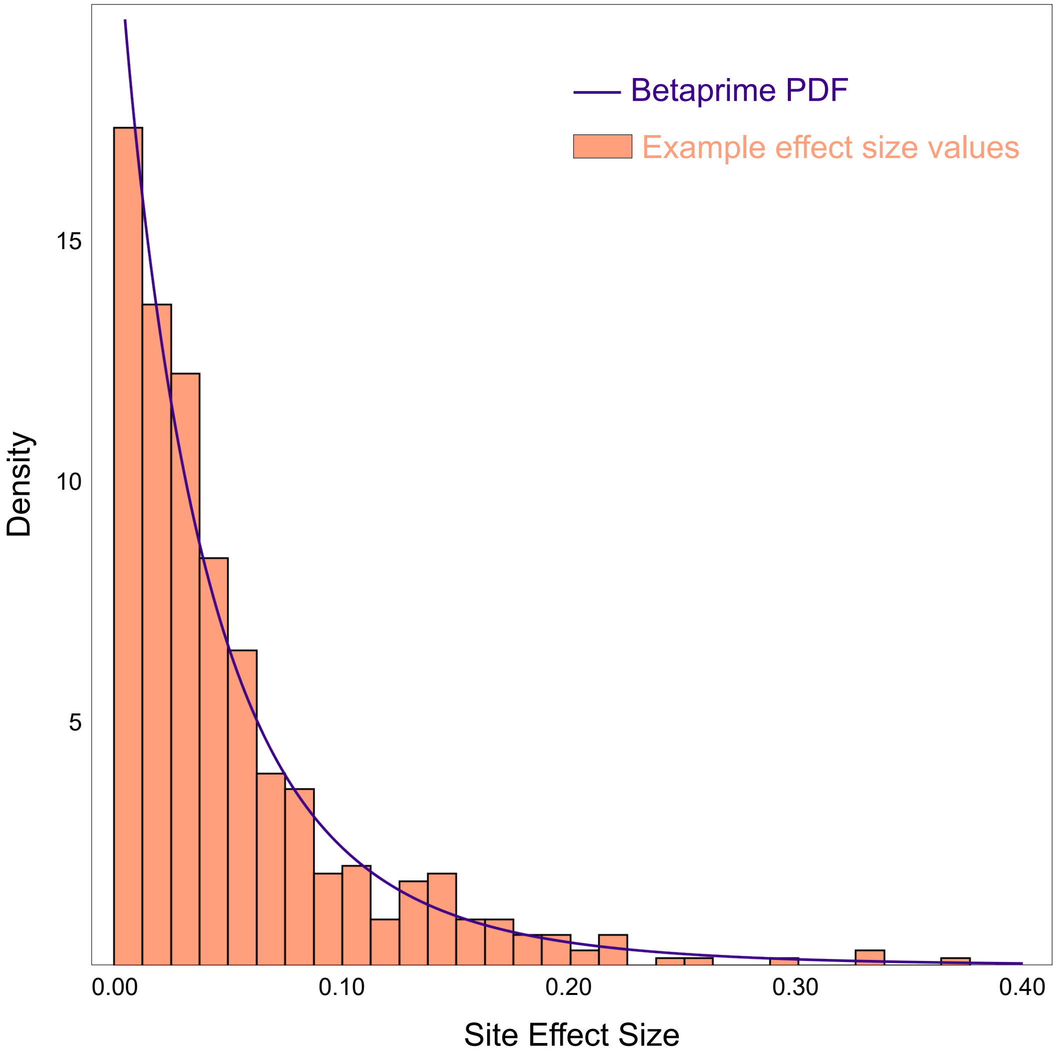

Figure S1: Distribution of Site Effect Sizes

Probability density function of betaprime distribution (Ayroles *et al.* 2015) from which site effect sizes are drawn (black line); example values of 500 sites with effect sizes drawn from the distribution from one particular run of our model.
