# Supplementary material for: Family-based selection: an efficient method for increasing phenotypic variability
Source: G3 (Bethesda). 2025 Jul 18;15(10):jkaf165. doi: 10.1093/g3journal/jkaf165 (PMC12506656; doi:10.1093/g3journal/jkaf165)
Supplement: jkaf165_Supplementary_Data [file jkaf165_Supplementary_Data.zip › Figure_S2_G3-2025-405909.pdf]

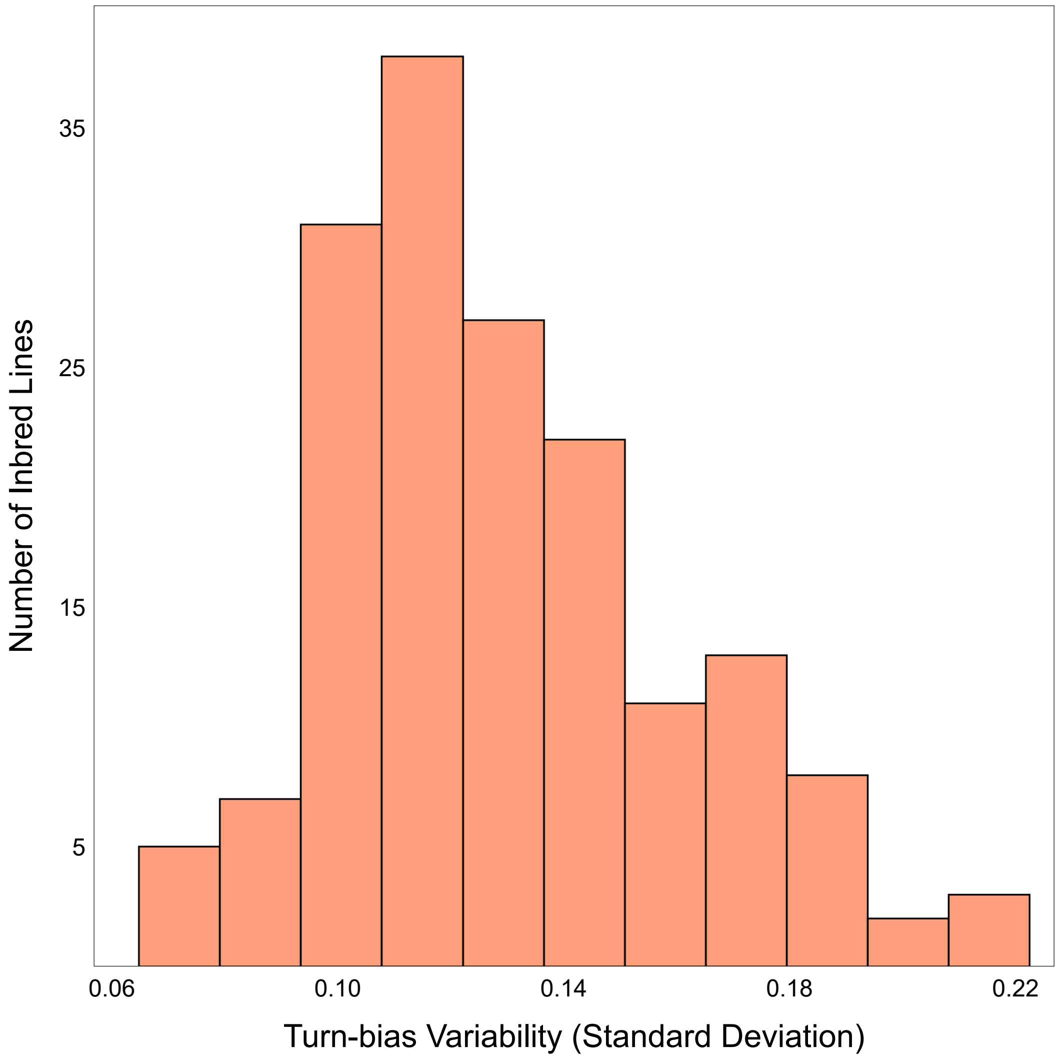

Figure S2: Empirical turn bias variability scores

Histogram of turn bias variability as measured across 167 inbred fruit fly lines (Ayroles *et al.* 2015). We mapped the summed variant effect sizes (and epistatic effects, if they were included in a model run) of individuals in our simulation to this distribution to determine the latent turn bias variability from which their individual turn bias phenotypes were drawn.
