# Supplementary material for: Family-based selection: an efficient method for increasing phenotypic variability
Source: G3 (Bethesda). 2025 Jul 18;15(10):jkaf165. doi: 10.1093/g3journal/jkaf165 (PMC12506656; doi:10.1093/g3journal/jkaf165)
Supplement: jkaf165_Supplementary_Data [file jkaf165_Supplementary_Data.zip › Figure_S4_G3-2025-405909.pdf]

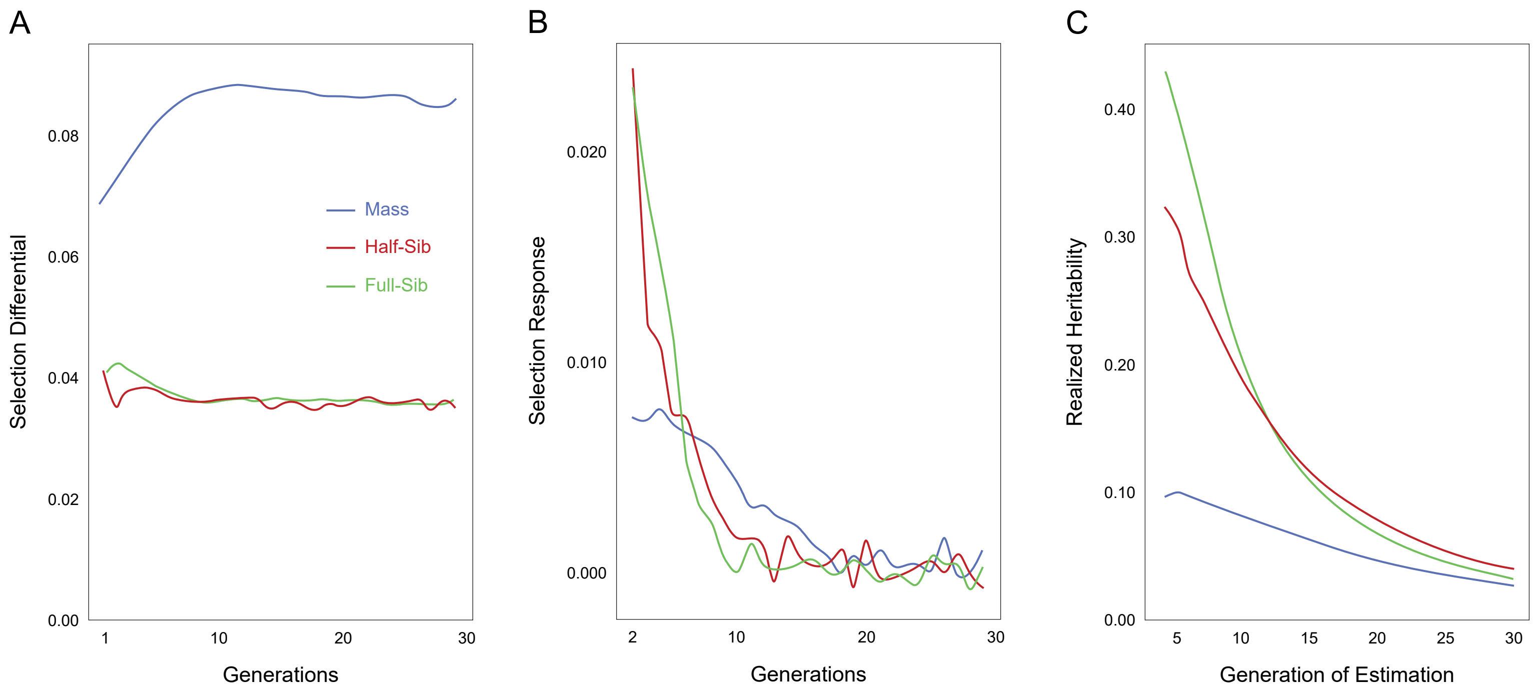

Figure S4: Selection differential, selection response and realized heritability across selection regimes

(A) Average value of selection differential ***S*** for the first 30 generations of selection, averaged over 100 model runs; (B) Average selection response ***R*** for the first 30 generations of selection; (C) Estimates of realized heritability h^2 at different time points across regimes
