# Supplementary material for: Family-based selection: an efficient method for increasing phenotypic variability
Source: G3 (Bethesda). 2025 Jul 18;15(10):jkaf165. doi: 10.1093/g3journal/jkaf165 (PMC12506656; doi:10.1093/g3journal/jkaf165)
Supplement: jkaf165_Supplementary_Data [file jkaf165_Supplementary_Data.zip › Figure_S6_G3-2025-405909.pdf]

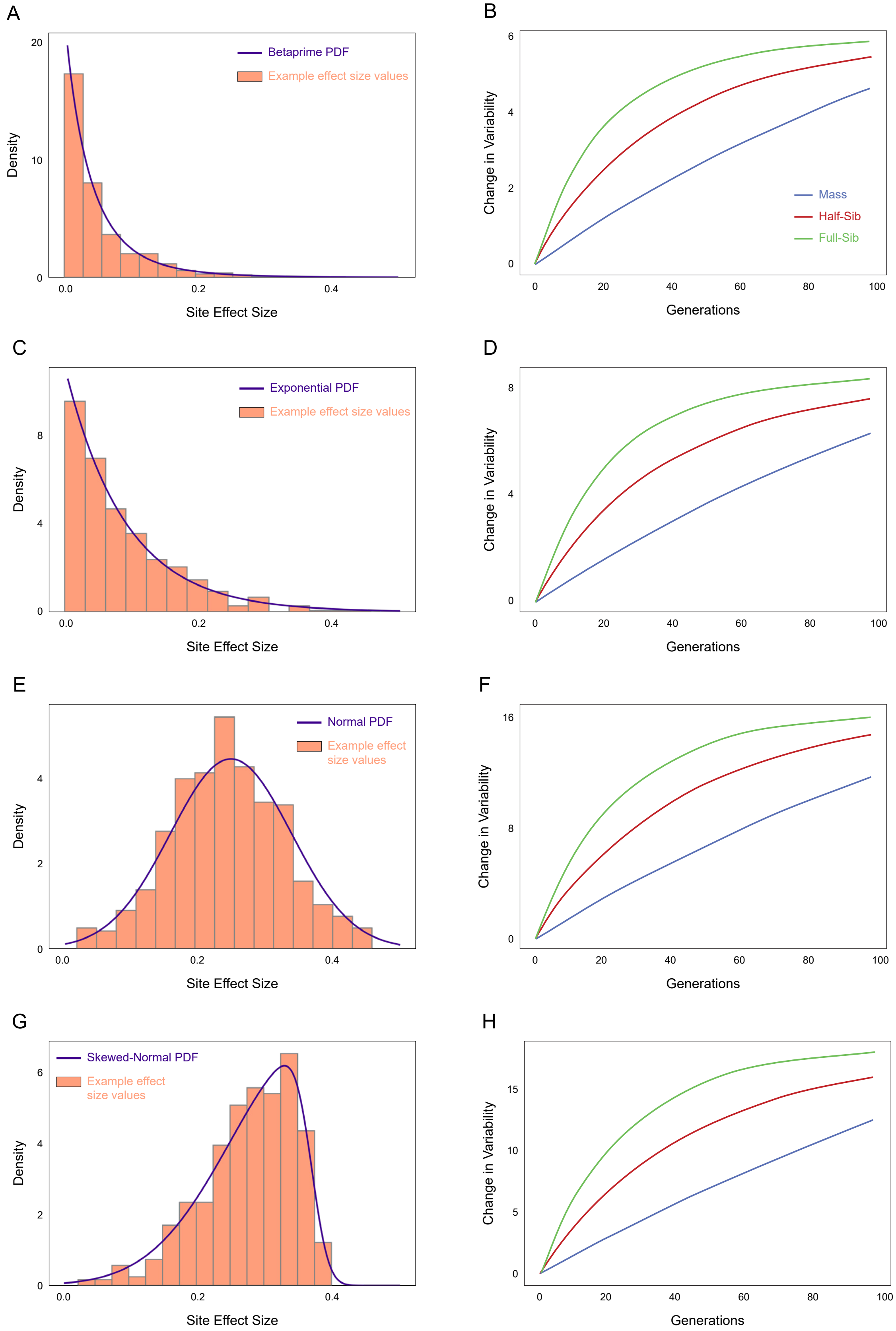

Figure S6: Effect of site effect size distribution on selection response

Probability density functions (PDFs) of distributions and corresponding example values of effect sizes for sites that contribute to variability for (A) Betaprime distribution, (C) Exponential distribution, (E) Normal distribution and (G) Skewed-Normal distribution; (B,D,F,H) Corresponding change in variability (i.e., standard deviation) as a function of number of generations of selection for various selection regimes. [$\boldsymbol{f(G_a) = G_a}$ for all cases]
