# Supplementary material for: Family-based selection: an efficient method for increasing phenotypic variability
Source: G3 (Bethesda). 2025 Jul 18;15(10):jkaf165. doi: 10.1093/g3journal/jkaf165 (PMC12506656; doi:10.1093/g3journal/jkaf165)
Supplement: jkaf165_Supplementary_Data [file jkaf165_Supplementary_Data.zip › Figure_S8_G3-2025-405909.pdf]

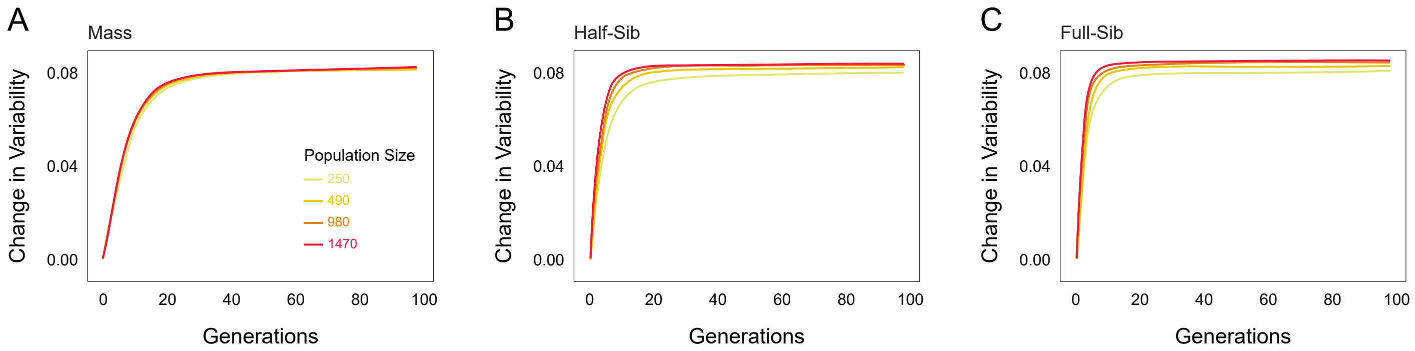

Figure S8: Effect of population size on selection response
Average change in variability as a function of the number of generations of selection with varying total population size in (A) Mass Selection; (B) Half-Sib Selection and (C) Full-Sib Selection
